# Exploring nationwide policy interventions to control COVID‐19 from the perspective of the rapid learning health system approach

**DOI:** 10.1002/lrh2.10363

**Published:** 2023-03-16

**Authors:** Ayat Ahmadi, Leila Doshmangir, Reza Majdzadeh

**Affiliations:** ^1^ Knowledge Utilization Research Center Tehran University of Medical Sciences Tehran Iran; ^2^ Social Determinants of Health Research Center Tabriz University of Medical Sciences Tabriz Iran; ^3^ Department of Health Policy & Management, Tabriz Health Services Management Research Center Tabriz University of Medical Sciences Tabriz Iran; ^4^ School of Health and Social Care University of Essex Colchester United Kingdom

**Keywords:** COVID‐19, health policy, health system, rapid learning

## Abstract

**Introduction:**

The health systems needed to improve their learning capacities during the COVID‐19 pandemic. Iran is one of the countries massively struck by the pandemic. This study aimed to explore whether and how the policy interventions made by Iran's policymakers at the national level to control COVID‐19, could improve the rapid learning characteristics of the health system.

**Methods:**

A guide to clarify rapid learning health system (RLHS) characteristics was developed. The guide was used by two independent authors to select the policy interventions that could improve RLHS characteristics, then, to analyze the content of the selected policy interventions. In each stage, results were compared and discussed by all three authors. Final results were presented based on different RLHS characteristics and the potential mechanisms of contribution.

**Results:**

Five hundred policy interventions were developed during the first 7 months of the outbreak. Thirty‐one policy interventions could potentially improve RLHS characteristics (6.2%). Two characteristics, such as the timely production of research evidence and the appropriate decision support were addressed by selected policy interventions. Policies, that could improve learning capacities, focused on decision‐maker groups more than user groups or researcher groups.

**Conclusions:**

Most of the developed policy interventions during the first months of the epidemic did not address the learning capacities of the health system. To improve health system functions, improving RLHS characteristics of the health system, especially in patient‐centered and data linkage characteristics, is recommended.

## 1 INTRODUCTION

To respond to the COVID‐19 pandemic, a wide range of policy interventions and programs were implemented across countries, either based on available evidence or previous experiences. Several policy measures have focused on improving the structure of the health system in addition to public health interventions such as travel restrictions, prohibitions on public meetings, and contact tracing.^1^, ^2^ The pandemic has made it clearer that health systems must be aware of the constantly shifting environment and ready to draw conclusions from their own data. Besides, the crisis put new demands on the health system in which people, media, and policymakers expected health research systems to have their questions answered outright.^3^

Rapid learning health systems (RLHS), an approach at the health system level that timely integrate internal data with updated evidence, as part of usual care, to provide high‐quality, safe, and more efficient services.^4^, ^5^

Evidence implies that learning health systems are effective in moving to provide better health services for communities.^6^ It is supported that the successful implementation of RLHS characteristics in health system structure will improve its functions in the health crises such as COVID‐19.^7^, ^8^ RLHS is concerned with the effective use of internal data and accessible evidence in health decision‐making, which is essential in times of health crisis, particularly in low‐ and middle‐income nations.^4^, ^5^ An efficient RLHS possesses different characteristics.^6^, ^9^ Lavis et al. introduced seven characteristics of an RLHS including engaging patients, digital capture, linkage and timely sharing of relevant data, timely production of research evidence, appropriate decision support, aligned governance, financial, and delivery arrangement, culturing rapid learning and improvement, and competencies for rapid learning and improvement.^9^ Health systems can improve these characteristics using various strategies. Based on Alliance for Health Policy and Systems Research report, health systems can intensify their learning capacities by learning via information, learning through deliberation, and learning through action. Health systems need to optimize the learning capacities of their stakeholders, such as people and patients, policymakers, and researchers.^5^

Iran is one of the countries massively struck by COVID‐19.^10^ From January 3, 2020, to July 26, 2021, there were 3 691 432 confirmed cases of COVID‐19, with 88 800 deaths in the country.^11^ In Iran, the Ministry of Health and Medical Education (MoHME) is responsible for the health system. Sixty‐two public medical universities under the stewardship of MoHME have the main role of providing health services for the populations they cover. Medical universities are also in charge of managing health research and delivering medical education. According to MoHME, the primary goal of the medical colleges' research system in recent years has been to increase the quantity of research publications.^12^ However, the COVID‐19 epidemic has brought up new arguments about the actual impact of health research in society.^3^

The establishment of a national entity (National Task Force for Fighting COVID‐19 [NTFFC]) was one of the primary initiatives to control COVID‐19 in Iran as the top national body to control COVID‐19 in the country. This entity was established by Supreme National Security Council to respond to the challenges of COVID‐19. Members of this committee include the representatives of relevant sections at the national level, such as MoHME, ministry of interior affairs, ministry of education, ministry of communication, military service, and police forces. The council's decisions were considered the main reference for other organizations to intelligently confront COVID‐19.^13^

We evaluated the policy interventions developed by Iran's NTFFC, during the first 7 months of the epidemic via RLHS’ perspective. The primary aim of the study was to find out whether and how the established policy interventions could improve the RLHS’ characteristics in Iran's health system.

## 2 METHODS

### 2.1 Study design

The document content analysis was the primary method for the data collection and analysis. All national policy interventions to control COVID‐19 during the first 7 months of the epidemic in the country were retrieved. To conduct the analysis, three main steps were followed as follows:

### 2.2 Developing a guide to clarify RLHS characteristics

A guide to analyzing the content of introduced policy interventions was developed based on RLHS characteristics, and the mechanisms of improvement,^5^, ^9^ In the developed guide each characteristic of RLHS was elaborated in a way that it becomes applicable for categorizing policy interventions. Moreover, a user should clarify the relation between each policy intervention and each RLHS characteristic, and provide answers to the following questions: What would be the main means of learning by this policy? How learning institutionalization would take place with this policy? Learning capacities of which group of stakeholders will be improved by this policy? What would be its main learning benefit and at which level of learning it may work?

### 2.3 Selection of the policy interventions which potentially contribute to RLHS

All national policy interventions^13^ were independently evaluated by two authors (LD & AA), to assess if they could potentially correspond with at least one of seven characteristics of RLHS. Policy interventions that could correspond to each characteristic of RLHS were selected by each author based on the presumed mechanism of implementation in the health system. Disagreements were settled by a conversation with the third author. The two writers' suggested methods for how certain policy actions may affect RLHS features were compared, debated, and refined by the three authors over the series of meetings.

### 2.4 Presentation of the results

To visualize a detailed description of findings, an Evidence, and Gap Map (EGM), was provided. The map shows selected policy interventions charted on the characteristics of RLHS and the potential mechanisms of their contribution. Rows show the potential way of correspondence of selected policy interventions to their relevant RLHS characteristic in relevant columns. In the cells, each mosaic indicates an RLHS‐related policy intervention. Click on a mosaic will give the content of the policy. The color of mosaics indicates the main probable benefit of the policy for relevant RLHS characteristics.

## 3 RESULTS

During the first 7 months of the epidemic, 500 policy interventions were made by Iran's NTFFC. The majority of the policies created had to do with limiting travel and transportation, organizing essential public services, controlling lockdowns and remote work, caring for patients, providing PPE for public officials and medical personnel, and introducing new rules for what information the public media could publish. Out of all policy interventions, 31 (6.2%) policies conformed to RLHS’ characteristics. Of these 31 policies, 12 policies (38%) were adaptable with more than one RLHS characteristic, and 6 were adaptable with more than 2 RLHS characteristics. The most RLHS‐contributing policy intervention was establishing a telephone hotline called “40, 30” (Forty, Thirty) (Table 1; decision no. 3) which was adaptable with four characteristics of RLHS, simultaneously. Timely production of research evidence 12 (38%), and the appropriate decision support 22 (48%) characteristics were more addressed by the policies. There was not any policy that could be linked to the competencies for RLHS characteristics. Table 1 shows selected policy interventions that were adaptable with at least one characteristic of RLHS. Regarding the probable means of learning, learning via deliberation was the most frequent (*n* = 15, 48.38%), followed by learning via information (*n* = 11, 35.48%) and learning via action (*n* = 5, 16.12%). Most selected policy interventions helped optimize learning capacities in the health care providers, managers, and policymakers groups (*n* = 21, 67.74%) while fewer policy interventions targeted researchers and data analysts (*n* = 7, 22.58%) and communities, citizens, and users (*n* = 3, 9.6%) groups. Table 2 presents mechanisms through which each selected policy intervention may improve RLHS. Supporting information represents the content of selected policies and the way that they may improve the RLHS characteristics.

**TABLE 1:** Policy interventions and their contribution to Rapid Learning Health System characteristics.

Row	Decision/policy	No. of repetitions in each characteristic	Rapid learning characteristics						
			People and patients partnership	Digital capture, linkage, and timely sharing of relevant data	Timely production of research evidence	Appropriate decision supports	Governance and financial arrangements	Culture of rapid learning and improvement	Competencies
1	Ministry of Health and Medical Education shall address various prospective scenarios concerning COVID‐19 and provide the results to the National Task Force for Fighting Coronavirus.	3			*	*		*	
2	The Ministry of Health and Medical Education is obliged to prepare all the required health instructions and communicate them to the administrative organizations.	3			*	*		*	
3	All organizations, in particular, the Ministry of Information and Communications Technology of Iran is obliged to cooperate in the quantitative and qualitative development of the 4030 hotline.	4	*	*	*		*		
4	The Ministry of Industry, Mine, and Trade should take the necessary measures to stabilize the commodity storage required for the end of the current year and the first months of the next year.	1				*			
5	The Ministry of Roads and Urban Development shall enforce all the health instructions and protocols provided by the Ministry of Health and Medical Education in all the airline, railway, land, and naval fleets within the country.	1				*			
6	The Ministry of Health and Medical Education, the Ministry of Interior, the Ministry of Roads and Urban Development, the Ministry of Cultural Heritage, Handicrafts and Tourism, the Islamic Republic of Iran Broadcasting (IRIB), and the Spokesperson of the Government of Iran quickly design flexible and motivational methods aimed to reduce Nowruz travels through the formation of advisory committees and provide the result to the National Task Force for Fighting Coronavirus.	2				*		*	
7	The Information and Education Committee (the Ministry of Culture and Islamic Guidance) prepares the instructions for taking legal action against producers and publishers who release fake news with the purpose of disconcerting public opinion.	1				*			
8	A special Working Group is constituted with the membership of the representatives of the Ministry of Health and Medical Education, the Ministry of Culture and the Islamic Guidance, and Islamic Republic of Iran Broadcasting (IRIB) to integrate and concentrate the sources of production and publication of news related to COVID‐19 at a national and international level.	2			*		*		
9	The Ministry of Health and Medical Education should establish a task force in the Deputy of Research and Technology to examine new COVID‐19 prevention and treatment initiatives.	1					*		
10	Working Group under the supervision of the Communication and Psychological Management Committee of National Task Force for Fighting Coronavirus is constituted with the membership of duly authorized representatives of the Secretary of National Security Supreme Council, the Prosecutor‐General of Iran, the Secretary of National Security Supreme Council, the chairman of the Islamic Republic of Iran Broadcasting, Minister of Culture and Islamic Guidance, Minister of Health and Medical Education, Minister of Information and Communications Technology of Iran, the General Staff of the Armed Forces, and with the responsibility of the secretary of Supreme Council of Cyberspace in the National Center for Cyberspace to reduce social harms, prevent the spread of rumors and unreliable news in social media, regulate the existing disorder in information sharing and immediately and effectively address the existing problems.	3			*	*		*	
11	To closely monitor the published news in all written and non‐written media and the Internet, a working group consisting of the Spokesperson of the Government of Iran, Minister of Culture and Islamic Guidance, the duly authorized representative of the Islamic Republic of Iran Broadcasting, and the judiciary hold daily meetings and monitor any violation of the defined policies and also take immediate and decisive measures proportionate to the severity of the violations. The scope of authority in this Committee will be provided in the subsequent meeting (21.03) by the Minister of Culture and Islamic Guidance.	2				*	*		
12	All the instructions and approvals of the National Task Force for Fighting Coronavirus should be implemented immediately and without delay. Any delays in implementation are construed as violations of the approvals provided by the National Task Force for Fighting Coronavirus and entail administrative prosecution.	2				*	*		
13	The procedure and protocol for sending the patients home, to hospitals, or temporary isolation facilities should be announced publicly and in a clear manner.	1				*			
14	The National Task Force for Fighting Coronavirus centrally makes decisions on issues related to the transportation of passengers and goods based on provincial requirements.	1					*		
15	The Ministry of Health and Medical Education is obliged to evaluate the implementation of the second phase of the social distancing plan by 04.04.	1				*			
16	The subsequent phases of the national COVID‐19 management are designed and provided at the national level and separately for each province based on the differences in healthcare, economic and social status of the provinces by provincial coronavirus management task forces and Ministry of Interior.	1				*			
17	The required plans and protocols for reopening educational facilities are reviewed based on the epidemic conditions in each province and submitted to the National Task Force for Fighting Coronavirus for approval.	1				*			
18	The Social and Security Committee provides a proposal on the status of religious ceremonies and holy shrines during the fasting month of Ramadan to decide in future meetings of the Task Force.	1				*			
19	The Ministry of Roads and Urban Development should communicate the health protocols applicable to all passengers traveling to Iran to all the air, land, and sea transportation services companies. The method of quarantine of passengers and its implementation is provided and implemented by the Ministry of Roads and Urban Development, and the Ministry of Health and Medical Education.	1				*			
20	The Ministry of Health and Medical Education is required to inform the people through official radio and television (IRIB) by 04.05 the criteria for determining the white, yellow, and red COVID‐19 state of each area to reopen certain mosques. Mosques and Friday prayers in the white areas will begin their activities by implementing the communicated protocols of the Ministry of Health and Medical Education in the middle of the month of Ramadan. The Ministry of Interior is responsible for conducting the necessary executive coordinating	2			*	*			
21	With the approval of the generalities in the plan for reopening schools and considering the three principles of a) maximum precaution for the health of students and their families; b) the quality of education; and c) no delay in the next academic year, the method and criteria of reopening the schools in low‐risk (white) cities will be reviewed until the next meeting, and implemented from 16.05.	2			*	*			
22	The Ministry of Health and Medical Education will implement the mechanism of effective monitoring of various groups and businesses through the cooperation and the presence of Basij, Law Enforcement Force, Ministry of Industry, Mine and Trade, Prosecutor, and Iran Chamber of Guilds to improve information dissemination, public education and encouraging approaches.	1				*			
23	All the governmental and semi‐governmental organizations, entities, military organs, law enforcement force, etc. are obliged to prepare their executive plans adopting an approach of combined coexistence and precaution about COVID‐19 and after obtaining the expert opinion of the Ministry of Health and Medical Education, submit the plan for the approval of the National Task Force for Fighting Coronavirus.	1				*			
24	All the administrative organizations should monitor the implementation of the approvals provided by the National Task Force for Fighting Coronavirus at all the administrative and social levels and submit a weekly report.	2		*	*				
25	According to the two indexes of the prevalence and incidence rates of the virus in each province and city, governors‐general and heads of universities of medical sciences, with the coordination of the Ministry of Health and Medical Education and the Ministry of Interior, are allowed to temporarily restrict some social activities affecting the disease, using a framework to be approved in the subsequent meeting, based on the current situation and the risk level of each occupation (high risk and medium risk.)		1			*			
26	In red provinces, the Provincial National Task Force for Fighting Coronavirus adopts the necessary measures to increase the monitoring of health instructions and protocols in various spheres.	1				*			
27	The Security, Disciplinary and Social Committee is obliged to provide weekly recommendations as necessary, about improving the quality of protecting the prisoners and the number of prisoners allowed to be kept in each prison, and announce it to the judiciary, based on the evaluation of reports on monitoring the communicated protocols throughout the country and reviewing of the situation.	3			*	*	*		
28	According to the report of the Provincial Task Force for Fighting Coronavirus on the current situation and future forecast of the trend of the disease, with the approval of the Security, Social and Disciplinary Committee of the National Task Force for Fighting Coronavirus, the Minister of Interior and the Minister of Health and Medical Education will propose the necessary measures to reduce the spread of the disease or increase smart reopenings in each case at the province, county, and city levels. These measures will be implemented after the approval of the President.	1				*			
29	The executive methods of monitoring include public (collective) monitoring, organizational monitoring, self‐reporting, and the use of governmental capacities and active systems in social media.	1	*						
30	According to the necessity of the planning concerning compatibility with COVID‐19, all organizations, including governmental, non‐governmental, semi‐governmental, military, judicial, and private entities are required to complete a checklist provided by the Deputy Minister of Health and Medical Education.	1			*				
31	Information about the statistics of (COVID‐19) deaths, patients, medicines, health issues, closures or restrictions on occupations, businesses, and facilities, and other matters related to COVID‐19 is only determined through official sources and in coordination with the Information Committee.	3		*	*		*		
	Total		2	3	12	22	8	4	0

**TABLE 2:** The probable ways that each RLHS‐related policy or decision may improve RLHS

Policy number (see Table 1)			1	2	3	4	5	6	7	8	9	10	11	12	13	14	15	16	17	18	19	20	21	22	23	24	25	26	27	28	29	30	31	Total
Means of learning		Learning through information		*	*					*	*	*								*						*	*			*	*		*	11
		Learning through deliberation					*	*	*				*		*	*	*				*	*	*	*	*			*	*			*		15
		Learning through action	*			*								*				*	*															5
Building learning health system	Institutionalizing learning	Institutionalize learning through information.		*			*				*					*		*		*		*	*	*	*	*	*	*	*	*		*	*	17
		Institutionalize deliberative learning	*		*	*		*	*	*		*	*				*		*		*										*			12
		Institutionalizing experiential learning												*	*																			2
	Optimizing learning capacities	Communities, citizens and users							*																				*	*				3
		Care providers, managers and policymakers	*	*	*	*		*		*		*	*	*	*	*	*	*	*		*	*		*	*			*			*		*	21
		Researchers and analysts					*				*									*			*			*	*					*		7
Benefits		System functions	*	*	*					*		*	*	*		*	*				*	*	*	*	*	*		*	*	*	*		*	21
		Adoptability and innovation				*	*	*			*				*			*		*							*					*		8
		Self‐reliance							*										*															2
Learning levels		Improved system functions							*					*												*	*			*	*	*		7
		Adaptivity and innovation									*				*	*	*	*	*	*			*						*					9
		Organization‐cross‐organization	*	*	*	*	*	*		*		*	*								*	*		*	*			*					*	15

## 4 DISCUSSION

The content of policy interventions introduced to control COVID‐19 in Iran at the national level were evaluated to examine whether they could improve the RLHS characteristics of the national health system. In contrast to policy interventions aimed at enhancing learning capacity and data linkage in the health system, public health policy initiatives, such as limitations on travel and transit and controlling lockdown norms, were implemented significantly more often. Among those policy interventions which could improve RLHS characteristics, those that were related to the timely production of research evidence and providing appropriate decision support characteristics were addressed more frequently.

People and patients' partnership in health decision‐making is one of the main characteristics of RLHS. Besides, it was shown that for the successful implementation of public health interventions, people, and patient partnership plays a crucial role.^14^ Our findings showed that policy interventions targeting the improvement of community engagement were not considered during the first months of COVID‐19 in the country. Developed policy interventions during the same time, were, in essence, unilateral and not able to improve the people and patients' partnership in the process of decision‐making.

Considering the urgency of questions, and new challenges that health systems faced up to during the COVID‐19 pandemic, obtaining well‐processed data was an essential source of information for decision‐makers.^15^ Despite producing a huge amount of data and information by various stakeholders during COVID‐19, and introducing some opportunities for data linkage in the country,^16^, ^17^ our findings showed that policy interventions concerning the optimal use of data were not made frequently. Instead of enhancing data linking and data capturing capabilities, associated policy actions have placed a greater emphasis on the daily reporting of new cases and fatalities. The real number of illnesses and fatalities is believed to have been substantially greater than the daily statistics, despite the fact that they served as the primary criterion for mandating or loosening restriction measures.^18^ Moreover, the main reason for the fewer decisions about digital data capturing and data linkage originated in the limited infrastructure capabilities of health information systems.^19^

Based on our findings, building relationships among and across different sectors in and out of the health system was a big concern for Iran's national policymakers in the first months of the epidemic. We considered such policy interventions as policies that support the learning capacities, and data linkage of the health system.^7^, ^9^ However, the study findings showed that there was no policy that could be considered a significant improvement for the culture and competencies of RLHS, as they were defined^9^ during the study time.

The unknown nature of the crisis, limited financial support, and limitations in inter‐sector collaboration caused many challenges during the implementation of the policy interventions.^20^ However, during the time of the implementation of policy interventions, daily new detected cases decreased from more than 35 000 in February 2020 to about less than 150 cases in the middle of June 2020.^11^ While it is unclear how much of this significant drop can be attributable to those efforts,^21^ the daily notifications of new cases and fatalities did contribute to a little amount of public confidence being restored. Public trust was considerably damaged because of main governmental public media tried to downplay the situation in the first weeks of the pandemic.^22^ Another effect of implemented policy interventions was modifying the centralized nature of the health system. Most of the decisions and executive orders, that were taken centrally before and during the first weeks of the epidemic, were left to local authorities after a month of NTFFC commencing.^23^

## 5 CONCLUSION

Most of the developed policy interventions during the first months of the epidemic in Iran did not address the learning capacities of the health system. Reconfiguration of the health system functions by considering the characteristics of RLHS can improve the health system's deliveries and services, especially in response to emergency situations like COVID‐19. To enhance the operations and outputs of the health system, policy interventions that address the relationship between people and patients, data linkage and data collection, as well as cultural and RLHS competences, must be taken more seriously.

## CONFLICT OF INTEREST

The authors have no conflicts to disclose.

## ETHICS STATEMENT

This study was approved by the ethics committee of Tehran University of Medical Sciences, Tehran, Iran (Approval No: IR.TUMS.NIHR.REC.1399.017).

## Supporting information

Supporting Information.
