# Supplementary material for: Exploring nationwide policy interventions to control COVID‐19 from the perspective of the rapid learning health system approach
Source: Learn Health Syst. 2023 Mar 16;8(1):e10363. doi: 10.1002/lrh2.10363 (PMC10797566; doi:10.1002/lrh2.10363)
Supplement: Supplementary file 1 — Supporting Information. [file LRH2-8-e10363-s001.html]

EPPI-Mapper


X

- Filters
- Hide Headers
  Show Headers
- Fullscreen
  Exit Fullscreen
- About
- Submit a Study
- View Records

Exploring Iran's policy interventions developed to control COVID-19 from the perspective of the rapid learning health system approach

Generated using v.2.1.0 of the EPPI-Mapper
powered by EPPI Reviewer
and created with


by the
Digital Solution Foundry team.
